# How blindness shapes personality: a neuro-ecological account

**DOI:** 10.3389/fnins.2026.1759728

**Published:** 2026-04-10

**Authors:** Xue Zhang, Qiqi Dong, Yang Liu, Jine Xu, Liyuan Lin, Yi Ji, Yu Zhang, Zhen Zhao, Zhongyu Chang, Luli Wei, Xin Li, Yun Luo, Xinglong Fu, Yu Liu, Chong Liu, Hao Ding, Wen Qin

**Affiliations:** 1 Department of Radiology, Tianjin Key Lab of Functional Imaging, Tianjin Institute of Radiology and State Key Laboratory of Experimental Hematology, Tianjin Medical University General Hospital, Tianjin, China; 2 Department of Radiology, Baoding No.1 Central Hospital, Baoding, China; 3 Department of Radiology, Beijing Friendship Hospital, Capital Medical University, Beijing, China; 4 School of Medical Imaging, Division of Medical Technology, Tianjin Medical University, Tianjin, China

**Keywords:** visual impairment, personality, social support, mobile phone, magnetic resonance imaging, neuro-ecological interaction, contextual environment, neural plasticity

## Abstract

**Introduction:**

The established link between personality and psychological well-being underscores the need to understand how major life changes, such as vision loss, reshape an individual’s disposition. While previous research has produced inconsistent findings, the roles of concurrent environmental factors and underlying neural mechanisms have remained largely unexplored.

**Methods:**

This study employed an integrated neuro-ecological framework to investigate how blindness influences personality. We recruited 46 blind participants and 41 sighted controls, who completed comprehensive assessments including the NEO-Five-Factor Inventory, social and lifestyle questionnaires, and multimodal neuroimaging, including structural magnetic resonance imaging (MRI), diffusion MRI, and resting-state functional MRI.

**Results:**

Blind participants showed higher agreeableness, extraversion, and conscientiousness, while reduced neuroticism compared to sighted controls, and these personality trait differences were attenuated after accounting for trait anxiety. These differences were partially mediated by increased perceived social support from friends. Furthermore, mobile phone usage habits showed an interaction with blindness on personality traits. Neuroimaging identified both shared and vision-specific neural correlates of personality. For instance, blindness-related changes in white matter integrity of the anterior thalamic radiation and forceps minor mediated the reduction in neuroticism. Moderated mediation models further revealed that the strength of these neural pathways was regulated by environmental factors, such as social support and mobile phone self-control.

**Discussion:**

Collectively, these results indicate that personality patterns in blindness are a dynamic process involving the interplay of neural plasticity and environmental modulation, rather than a direct consequence of sensory loss alone.

## 1 Introduction

Vision profoundly influences how individuals perceive, interpret, and navigate their surroundings, and its loss presents profound challenges that extend beyond sensory experience to affect quality of life, cognitive processes, and mental well-being (Burton et al., 2021; Steinmetz et al., 2021). Personality—a stable configuration of an individual’s thoughts, emotions, and behaviors—shapes how people respond to major life changes and adversity (John et al., 2008). The Big Five model, encompassing neuroticism, extraversion, openness, agreeableness, and conscientiousness, has emerged as a robust and cross-culturally validated framework for personality assessment (Schmitt et al., 2007). Its concise design and consistent psychometric properties have allowed large-scale comparisons across diverse populations (Awwad and Al-Aseer, 2021; Mammadov, 2022; Kumar et al., 2023). Moreover, personality traits such as neuroticism and agreeableness have been found to be closely associated with mental disorders and show similar trait profiles, suggesting that personality may partly reflect an individual’s mental health status (Kotov et al., 2010; Gupta et al., 2024). However, a consensus on personality differences associated with blindness has yet to be reached. Some studies report no meaningful differences between blind and sighted groups (Zahran, 1965; Warren, 1984; Harrington and McDermott, 1993), whereas others identify notable differences using different test batteries (Adrian et al., 1982; Klinkosz et al., 2006; Papadopoulos et al., 2013; Gerc, 2018). These inconsistencies may partly arise from the considerable heterogeneity among blind individuals—particularly in terms of onset (congenital vs. acquired), duration, residual vision, and etiology—each of which may differentially shape personality development. Furthermore, variability in assessment tools and insufficient control of contextual factors may also contribute to inconsistent findings across studies.

Personality does not exist in isolation; it emerges from a rich interplay of biological and environmental influences (Caspi et al., 2005). Social support, perceived social status, lifestyle habits, and technology use are recognized contributors to personality development. Stronger perceived social support is generally associated with higher agreeableness, extraversion, and conscientiousness, and lower neuroticism (Udayar et al., 2020; Hill et al., 2024). Similarly, individuals with lower subjective social status tend to exhibit higher neuroticism and lower traits reflecting social engagement and regulation (Chapman et al., 2010; Bucciol et al., 2015). Modern lifestyle factors, such as sleep quality, dietary patterns, and mobile phone use have also been linked to personality traits (Gao et al., 2020; Siegrist et al., 2022; Guerreiro et al., 2024). Extensive research has demonstrated that visual loss profoundly impacts the surrounding environment of blind individuals (Marques et al., 2021; Wang et al., 2022). However, most studies on the personality of blind individuals have not accounted for the concurrent influence of these contextual environmental factors. Neglecting these accompanying changes hinders a comprehensive understanding of the multifaceted processes that guide personality formation in blind people.

Parallel research in cognitive neuroscience shows that brain morphology and function are associated with individual differences in personality. Research in sighted populations has identified correlations between personality traits and the structure and function of various brain regions, including the inferior temporal cortex, superior frontal cortex, and white matter tracts like the anterior thalamic radiation (Lewis et al., 2018; Xue et al., 2023; Zhang et al., 2023). Functional studies similarly link intrinsic brain activity to personality-related emotional and social processing (Simon et al., 2020; Wang et al., 2025). Notably, some of these regions overlap with brain reorganization by visual deprivation. Blindness is known to be associated with extensive neuroplasticity, not only within the visual cortex (Yu et al., 2008; Jiang et al., 2009), but also in circuits supporting other sensory and higher-order cognitive functions like emotional regulation and social cognition (Liu et al., 2007; Rakesh and Whittle, 2021; Salinas et al., 2021). These overlapping neural transformations raise the possibility that large-scale brain reorganization, driven by both sensory deprivation and subsequent environmental adjustments, could modulate the neural circuits associated with personality (Cai et al., 2020; Chen et al., 2022).

**Figure 1:**
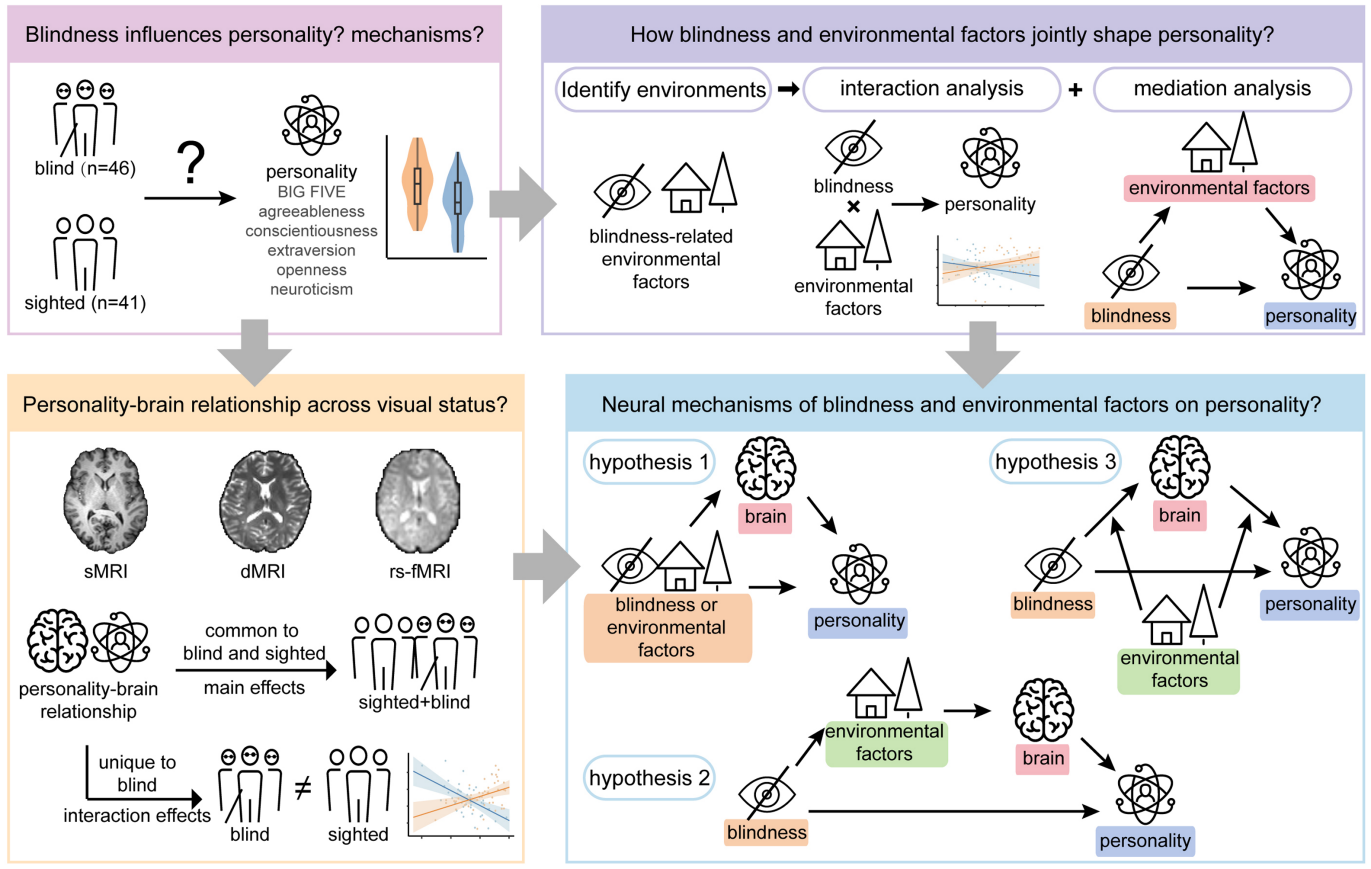
Flowchart of the study design. sMRI, structural magnetic resonance imaging; dMRI, diffusion magnetic resonance imaging; rs-fMRI, resting-state functional magnetic resonance imaging.

Despite growing evidence, studies that simultaneously incorporate personality, sensory deprivation, environmental contexts, and brain correlates remain scarce. The present work introduces a neuro-ecological framework to address this gap by integrating multidimensional environmental factors and multimodal magnetic resonance imaging (MRI) phenotypes to examine the impact of blindness on personality subscales. This study aims to identify the specific Big Five personality traits associated with blindness and to elucidate the underlying environmental and neurobiological mechanisms driving these associations. Based on the identified associations, we propose the following hypotheses: (1) The association between blindness and personality is shaped by environmental contexts through two pathways: (a) environmental contexts mediate the association between blindness and personality traits; (b) environmental contexts moderate the association between blindness and personality traits (interaction). (2) Brain structure and function may integrate these effects through the three candidate pathways: (a) blindness and environmental contexts are each independently associated with personality, with effects mediated by brain structure and function; (b) the relationship between blindness and personality is serially mediated by environments and brain phenotypes; (c) environmental contexts moderate the pathway linking blindness, brain alterations, and personality. In addition, we further assessed the effects of mental status (depression/anxiety) on the associations between blindness and personality traits. Aspects related to blindness were also considered to account for potential heterogeneity among individuals with blindness. The flowchart of the study is presented in Figure 1.

## 2 Materials and methods

### 2.1 Participants

Forty-six blind participants (23 males; mean ± SD = 44.67 ± 9.84 years) and 41 sighted participants (23 males; 43.07 ± 10.41 years) took part in the study. Blind participants met the 1973 World Health Organization criteria for complete blindness (best-corrected visual acuity < 0.05 or visual field < 10°) and were capable of independent daily functioning within a community setting. Sighted controls had corrected visual acuity > 1.0 in each eye. Exclusion criteria were: severe neurological disorders (including cerebral infarction, intracranial hemorrhage, white matter lesions, brain tumors, or epilepsy); history of traumatic brain injury; major systemic diseases; current or past psychiatric hospitalization; substance dependence; and contraindications to MRI. Groups were comparable in age (t = 0.74, *p* = 0.463) and sex (χ^2^ = 0.32, *p* = 0.570). In the blind group, average age of blindness onset was 12.74 ± 13.05 years, and mean blindness duration was 31.50 ± 13.25 years. Detailed demographic information for each blind participant is provided in Supplementary Table 1, including sex, age of onset, duration, and etiology. The Medical Research Ethics Committee of Baoding No.1 Central Hospital approved all procedures, and all participants provided written informed consent.

### 2.2 Personality assessment

All participants completed the Chinese version of the NEO-Five-Factor Inventory (NEO-FFI) (Costa and McCrae, 1992), a 60-item Big Five personality traits questionnaire. This inventory has been repeatedly used in different Chinese populations and has been shown to have satisfactory validity and reliability (Yang et al., 1999; Zhang, 2003; Yu and Zhang, 2007). The Big Five personality traits include five dimensions: neuroticism, extraversion, openness, agreeableness, and conscientiousness. Neuroticism is related to emotional stability and self-regulatory abilities. Extraversion refers to the degree of extroversion and sociability. Openness reflects the willingness to accept new ideas, experiences, and possibilities. Agreeableness refers to friendly attitudes and behaviors towards others and society. Conscientiousness reflects one’s organization, reliability, and self-discipline. Each item was rated on a five-point Likert scale from 0 (strongly disagree) to 4 (strongly agree). After reverse scoring where appropriate, higher scores indicated stronger representation of the respective trait.

### 2.3 Environmental factors assessment

The contextual environmental factors were assessed for each participant, including perceived social support (family, friends, and significant others) (Zimet et al., 1988), subjective social status (Adler et al., 2000), sleep quality (Buysse et al., 1989), mobile phone addiction (inability to control craving, anxiety and feeling lost, withdrawal or escape and productivity loss) (Leung, 2008) and eating behaviors (Rosnah et al., 2013). Details regarding the questionnaires are provided as follows.

#### 2.3.1 Multidimensional scale of perceived social support (MSPSS)

The MSPSS measures perceived social support from a variety of sources, including three dimensions: family, friends, and significant others, which was designed by Zimet et al. (1988). This 12-item scale uses a 7-point Likert scale, with higher scores indicating greater perceived support.

#### 2.3.2 Chinese version of the subjective social status scale (CSSS)

Adapted from Adler et al.’s MacArthur Scale (Adler et al., 2000) and translated by Hu et al. (2012), the CSSS measures self-perceived socioeconomic position. The scale evaluates perceptions of family economic status and community environment across developmental stages, with higher scores reflecting elevated subjective social status.

#### 2.3.3 Pittsburgh sleep quality index (PSQI)

Sleep quality over the preceding month was assessed using the PSQI, originally developed by Buysse et al. (1989). The Chinese version, translated by Feng et al. (2005), comprises seven components scored 0–3, yielding a total score of 0–21. Higher scores indicate poorer sleep quality.

#### 2.3.4 Mobile phone addiction index (MPAI)

Mobile phone addiction was measured using Leung’s Chinese version of the MPAI (Leung, 2008). This 17-item instrument assesses four dimensions: inability to control craving (IC), anxiety/loss (AL), withdrawal/escape (WE), and productivity loss (PL), using a 5-point scale. Higher scores indicate greater addiction severity.

#### 2.3.5 Three-factor eating questionnaire-revised 21-item version (TFEQ-R21)

The TFEQ-R21, developed by Rosnah et al. (2013) and validated in Chinese populations by Zhang et al. (2016), evaluates maladaptive eating behaviors. This 21-item questionnaire demonstrates good psychometric properties, with higher scores indicating greater susceptibility to dysfunctional eating patterns.

### 2.4 Mental health assessment

#### 2.4.1 Beck depression inventory (BDI)

The BDI, developed by Beck et al. (1961), is one of the most widely used self-report instruments for assessing the severity of depressive symptoms. The inventory consists of 21 items, each rated on a 4-point scale ranging from 0 to 3, with total scores indicating minimal to severe depression. Higher total scores indicate greater severity of depressive symptoms.

#### 2.4.2 State–trait anxiety inventory (STAI)

The STAI, developed by Speilberger et al. (1983), is a widely used self-report measure for assessing anxiety. It comprises two 20-item subscales: (1) state anxiety index (SAI), which evaluates temporary and situational feelings of anxiety, and (2) trait anxiety index (TAI), which assesses an individual’s general tendency to experience anxiety. Each item is rated on a 4-point Likert scale, with higher scores indicating greater anxiety.

### 2.5 Brain MRIs data acquisition

All MRI data were acquired using a Philips Achieva 3.0 T scanner with an 8-channel head coil. (1) Structural MRI (sMRI): Acquired using a 3D turbo field echo sequence with parameters: TR (repetition time)/TE (echo time)/TI (inversion time) = 7.50/3.70/885 ms, FOV (field of view) = 256 × 227 mm, matrix = 256 × 227, flip angle = 8°, 188 sagittal slices (with 1 mm thickness and no gap). (2) Diffusion MRI (dMRI): Acquired using single-shot spin-echo echo-planar imaging with parameters: TR/TE = 7,000/101 ms, FOV = 256 × 256 mm, matrix = 128 × 128, flip angle = 90°, 48 axial slices (with 3 mm thickness and no gap). Acquisition included one b0 image and 32 diffusion-weighted directions (b-value = 1,000 s/mm^2^). (3) Resting-state functional MRI (rs-fMRI): Acquired using gradient-echo echo-planar imaging with parameters: TR/TE = 2,000/30 ms, FOV = 220 × 220 mm, matrix = 64 × 64, flip angle = 90°, 36 axial slices (with 3 mm thickness and 1 mm gap), time points = 180. Participants maintained eye closure without specific cognitive focus and reported compliance with instructions.

### 2.6 Structural MRI preprocessing

Standard voxel-based morphometry (VBM) analysis was conducted on sMRI data using Statistical Parametric Mapping 12 (SPM12)^1^ and the Computational Anatomy Toolbox 12,^2^ implemented in MATLAB R2016b (The MathWorks, Natick, MA). Preprocessing included brain tissue segmentation, DARTEL normalization (modulated by Jacobian determinants), and spatial smoothing with a 10 mm full-width at half-maximum (FWHM) Gaussian kernel. Finally, the absolute gray matter volume (GMV) was computed for each participant.

### 2.7 Diffusion MRI preprocessing

The dMRI data were processed with FSL6.0^3^ through the following pipeline: (1) Eddy-current and motion correction via FSL’s eddy toolkit with TOPUP for distortion correction and motion compensation (Andersson et al., 2003; Smith et al., 2004); (2) Brain extraction to remove non-brain tissues; (3) Tensor fitting using linear regression to generate white matter microstructural measures, including: fractional anisotropy (FA), radial diffusivity (RD), axial diffusivity (AD), and mean diffusivity (MD) maps; (4) Spatial normalization involving b0-to-sMRI affine registration followed by sMRI-to-MNI transformation using DARTEL deformation fields; (5) Spatial smoothing with an 8 mm FWHM Gaussian kernel.

### 2.8 Resting-state functional MRI preprocessing

Resting-state functional MRI data underwent preprocessing using SPM12 and DPABI V8.1 toolkits^4^ in MATLAB with the following pipeline: (1) Initial 10 volumes were discarded to ensure signal stabilization and scanner acclimatization. (2) Slice-timing correction addressed temporal acquisition discrepancies. (3) Rigid-body realignment compensated for inter-volume motion, with all participants demonstrating <2 mm/2° displacement. Frame-wise displacement (FD) was quantified. (4) Spatial normalization involved coregistering mean rs-fMRI to sMRI for affine transformation, followed by warping to MNI space via combined rfMRI → sMRI affine and sMRI → MNI DARTEL deformation fields, resampling to 3-mm isotropic resolution. (5) Nuisance regression removed 24 Friston motion parameters, FD > 0.5 volumes, and mean signals from global, ventricular, and white matter regions. (6) Band-pass filtering (0.01–0.10 Hz) attenuated low-frequency drift and high-frequency noise. (7) Spatial smoothing employed a 6 mm FWHM Gaussian kernel to enhance signal-to-noise ratio and statistical normality.

Regional homogeneity (ReHo) analysis computed Kendall’s coefficient of concordance (KCC) for each voxel’s time series relative to 26 adjacent neighbors (Zang et al., 2004) on unsmoothed preprocessed data. Resulting ReHo maps underwent whole-brain mean normalization followed by 6 mm FWHM Gaussian smoothing.

Additionally, amplitude of low-frequency fluctuations (ALFF) was calculated through the following procedure: (1) Nuisance-regressed but unfiltered rs-fMRI data underwent spatial smoothing with a 6 mm FWHM Gaussian kernel; (2) Fast Fourier transformation converted the smoothed time series to the frequency domain, with the square root of each voxel’s power spectrum computed and averaged across the 0.01–0.08 Hz frequency range to generate raw ALFF maps; (3) Each voxel’s ALFF value was standardized by division with the global mean ALFF value.

### 2.9 Statistical analyses

Scale scores were derived at the participant level. When more than one-third of items within a scale were missing or invalid, the corresponding scale score was set to missing. When one-third or fewer items were missing or invalid, missing values were imputed using the sample mean of the corresponding item prior to score calculation. Outliers were detected by using the interquartile range (IQR) method. Data points that fell below Q1–1.5 × IQR and above Q3 + 1.5 × IQR were considered outliers and treated as missing. For each analysis, only participants with non-missing data on all variables included in that analysis were retained.

#### 2.9.1 Inter-group differences in personality and environmental factors

Personality and environmental data were evaluated for normality using the Shapiro–Wilk test. Two sample *t*-tests were carried out for inter-group differences if data followed normal distribution, and Welch’s *t*-test was used if variances were not homogeneous. Wilcoxon rank-sum test was performed when the data follow non-normal distribution. Effect sizes for t-tests were calculated as Cohen’s d. For Wilcoxon rank-sum tests, effect sizes were calculated as r. Ninety-five percent confidence intervals were reported for all effect sizes. All data were adjusted for age and sex using linear regression before statistics. Multiple comparisons were adjusted using false discovery rate (FDR) (corrected *p* < 0.05, two-sided).

#### 2.9.2 Relationships between anxiety/depression, personality traits and blindness

First, we examined the relationships between personality traits and mental health status (BDI, SAI, TAI) using Pearson or Spearman partial correlation depending on the normality of data (Shapiro–Wilk test). These correlations were calculated separately in the full sample, as well as in the blind and sighted subgroups, separately. In addition, to assess the effect of mental health status on group differences in personality traits, mental health scores were regressed out from each personality measure using linear regression models. Group comparisons were then performed on the resulting residuals using the same analytical approach described above. All analyses were adjusted for age and sex, and corrected for multiple comparisons using the FDR method (corrected *p* < 0.05, two-sided).

#### 2.9.3 Association between MRI-derived phenotypes and personality/blindness

Whole-brain voxel wise general linear models (GLM) assessed (1) main effects of personality traits (X) on MRI-derived phenotypes (Y) across all participants and (2) interaction effects between personality and blindness on MRI-derived phenotypes using SPM12,^5^ controlling for age and sex. GMV statistics additionally controlled for total intracranial volume (TIV). Clusters were considered significant at cluster-wise *p* < 0.05 using family-wise error (FWE) correction with an initial voxel-level threshold of *p* < 0.001. Regions of interest (ROI) were defined around peak voxels (6 mm radius) for GMV and rs-fMRI phenotypes (ReHo and ALFF), or from significant fiber clusters (>20 voxels) for dMRI using Johns Hopkins University White-Matter Tractography Atlas (thresholded at 25%). Mean values per ROI were extracted for subsequent analyses. To explore whether these personality-associated brain phenotypes are remodeled after blindness, we performed between-group comparisons in ROI-wise brain phenotypes between blind and sighted subjects, using the same methods as for personality and environmental inter-group comparisons (two-sided *p* < 0.05, FDR correction).

#### 2.9.4 Mediation and interaction effects of blindness and contextual environments on personality

Since both blindness and their contextual environments are potential factors for personality, we hypothesized that these two types of factors may relate to personality traits via two pathways: (1) A mediation pathway, in which the relationship between blindness and personality is mediated by environmental factors. (2) An interaction pathway (moderation), where the effect of blindness on personality is dependent on specific environmental contexts, meaning it only manifests in subgroups with certain levels of environmental exposure.

To test for mediation, PROCESS macro v4 for R (Hayes, 2022) was applied, with blindness as independent variable, environmental contexts as mediators, and personality traits as outcome variable, accounting for age and gender. The significance of the indirect effect was assessed using 95% percentile confidence intervals (CI), generated from 1,000 bootstrap samples. All variables were standardized prior to mediation analysis, and standardized coefficients are reported. We also calculated the proportion of mediation (MP) for each significant model. To test for interaction, a GLM with personality traits as the dependent variable, blindness and contextual environments, and their interaction term as independent variables was applied, accounting for age and gender. A statistically significant regression coefficient for the interaction term indicated an interaction (two-sided, *p* < 0.05, uncorrected).

#### 2.9.5 Neural pathways underlying the associations of blindness and contextual environments with personality

To investigate the neural pathways by which blindness and contextual environments relate to personality, we hypothesized three potential associative pathways in which the brain may participate: (1) Simple mediation pathway: brain structural and functional measures are examined as mediators linking blindness and contextual environments separately with personality traits. (2) Serial mediation pathway: contextual environmental factors and brain measures are modeled as sequential mediators in the association between blindness and personality traits (i.e., blindness → contextual environments → brain → personality). (3) Contextual environmental factors are tested as moderators of the pathways linking blindness, brain measures, and personality traits, specifically moderating either the blindness → brain association or the brain → personality association. We conducted mediation analyses using ROIs identified from the main effects of personality. The Index of Moderated Mediation (IMM) was used to estimate whether the moderated mediation effect was significant, and the indirect effect was estimated separately at low (16%) and high (84%) levels of moderators if IMM is significant (95% percentile CI).

#### 2.9.6 Sensitivity analysis

To examine the effects of different blindness conditions, two sensitivity analyses were performed. First, to examine differences in personality and contextual environments as a function of age at onset, participants with blindness were primarily classified into early-onset (0–6 years) and late-onset (>6 years) groups; additional cutoff of 10 years (the WHO-defined onset of adolescence) were conducted as an alternative threshold. A one-way analysis of covariance (ANCOVA) was conducted for normally distributed data, and a Kruskal–Wallis test was used for non-normal data to compare group differences (early blind, late blind, healthy control), controlling for age and sex effects (two-sided, *p* < 0.05, uncorrected). *p* values from the three *post hoc* pairwise comparisons were adjusted using the FDR correction (corrected *p* < 0.05, two-sided). Additionally, to assess whether the age of onset and duration of blindness were associated with personality and other environmental factors, Pearson’s or Spearman’s correlation analyses were conducted depending on data normality, without controlling for age and sex (two-sided, *p* < 0.05, uncorrected). All normality tests were assessed using the Shapiro–Wilk test.

## 3 Results

### 3.1 Differences in personality and contextual environments between blind and sighted individuals

**Figure 2:**
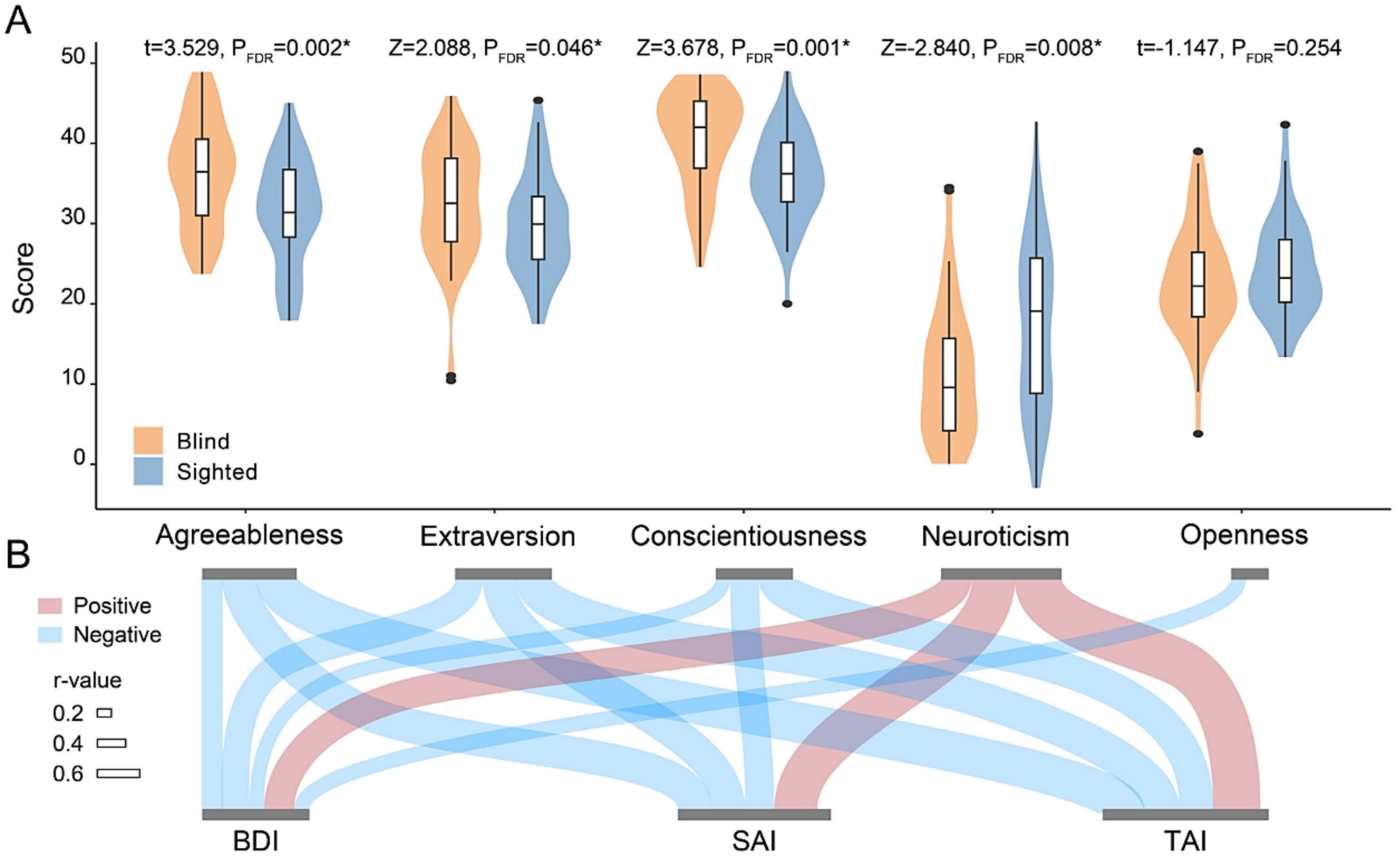
Personality differences between blind and sighted people and association with mental health. Sample size = 87 (cases = 46). The results were adjusted for age and sex. Statistical significance was adjusted by false discovery rate (FDR) correction (pFDR < 0.05). **(A)** Personality differences between blind and sighted people. Asterisks indicate statistical significance with pFDR < 0.05. **(B)** Partial correlations between personality traits and depression/anxiety measures in the full sample. Edge widths reflect the strength of the correlation coefficients, with red edges indicating positive correlations and blue edges indicating negative correlations. Only significant edges (pFDR < 0.05) are shown. BDI, beck depression inventory; SAI, state anxiety index; TAI, trait anxiety index.

Relative to sighted participants, blind individuals scored higher on agreeableness (d = 0.76 [0.32, 1.20], t = 3.53, pFDR = 0.002), extraversion (r = 0.22 [0.03, 0.42], Z = 2.09, pFDR = 0.046), and conscientiousness (r = 0.39 [0.21, 0.57], Z = 3.68, pFDR = 0.001), and lower on neuroticism (r = 0.30 [0.10, 0.50], Z = −2.84, pFDR = 0.008). No significant difference in openness was observed (d = −0.25 [−0.68, 0.18], t = −1.15, pFDR = 0.254) (Figure 2A). Blind group reported significantly higher MSPSS_Fri (r = 0.29 [0.08, 0.48], Z = 2.60, pFDR = 0.009). They also showed lower MPAI_IC (r = 0.28 [0.05, 0.47], Z = −2.54, pFDR = 0.011) but higher MPAI_WE (r = 0.47 [0.27, 0.63], Z = 4.30, pFDR < 0.001). Other environmental factors, including PSQI, TFEQ, and CSSS, did not differ significantly (Supplementary Table 2).

### 3.2 Relationships between personality traits and anxiety/depression

Partial correlation analyses revealed that, in the full sample, neuroticism was positively associated with depressive symptoms (BDI: r = 0.46, pFDR < 0.001) and both state and trait anxiety (SAI: r = 0.66, pFDR < 0.001; TAI: r = 0.73, pFDR < 0.001). Agreeableness, extraversion and conscientiousness were negatively correlated with depression and anxiety measures (r ranged from −0.62 to −0.22, all pFDR < 0.05). Openness was significantly associated with depressive symptoms (BDI: r = −0.23, pFDR = 0.039), but not with anxiety measures (SAI: r = −0.21, pFDR = 0.052; TAI: r = −0.13, pFDR = 0.245) (Figure 2B). In the blind and sighted subgroups, the correlation patterns were largely consistent with the full sample (Supplementary Figures 1,2).

### 3.3 Influence of anxiety/depression on the association between blindness and personality traits

After controlling for BDI, intergroup differences in agreeableness, extraversion, neuroticism, and conscientiousness remained significant (FDR-corrected *p* < 0.05), suggesting that depressive symptoms did not account for the association between blindness and these personality dimensions. After controlling for SAI, only the difference in conscientiousness remained significant (t = 3.16, pFDR = 0.009). No intergroup differences were observed after controlling for TAI or for all mental health measures (BDI, SAI, and TAI) (all pFDR > 0.05), indicating that anxiety—particularly trait anxiety—may interact with the relationship between blindness and personality traits (Supplementary Table 3).

### 3.4 Mediation and interaction effects of blindness and contextual environments on personality

**Figure 3:**
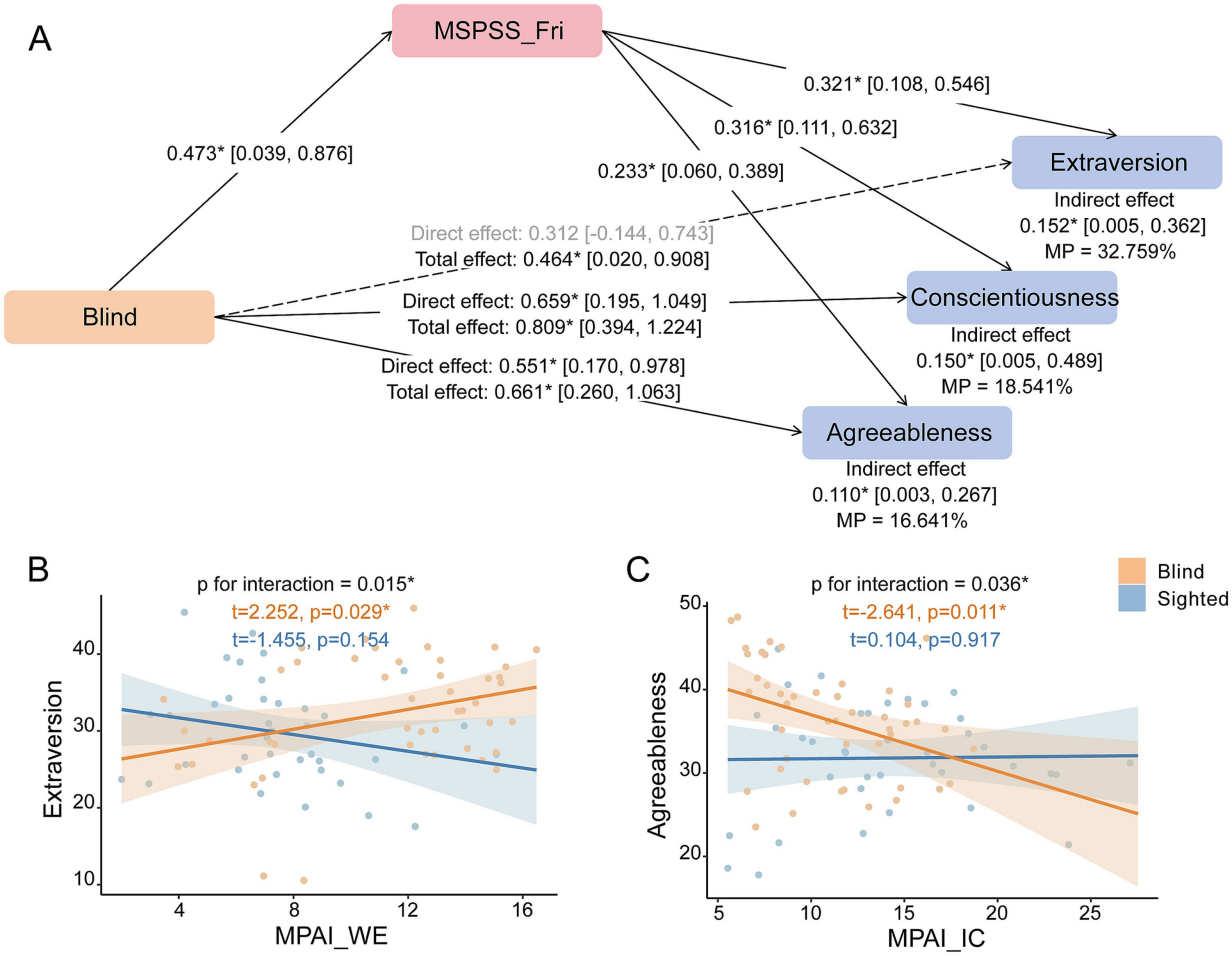
Mediation and interaction effects of blindness and contextual environments in relation to personality. **(A)** Shows the mediation findings. Sample size = 80 (cases = 46). Significance of mediation effects is estimated by 95% percentile confidence intervals using 1,000 bootstraps. Asterisks + solid lines + black font indicate significant pathways, while dashed line with gray font indicate non-significant pathways. **(B)** and **(C)** Show the interaction findings between MPAI subscale and blindness. Sample size = 84 (cases = 46). Asterisks represent significant effects (puncorrected < 0.05). All results were adjusted for age and sex. Abbreviations: MP, mediation proportion; MSPSS_Fri, perceived social support from friends; MPAI_WE, withdrawal and escape for mobile phone use; MPAI_IC, inability to control craving for mobile phone use.

Blindness and blind-related environmental factors collectively relate to personality, showing both interaction and mediation relationships (Figure 3). Specifically, mediation analysis revealed that MSPSS_Fri mediated the relationship between blindness and agreeableness (standardized indirect effect = 0.110 [0.003, 0.267], MP = 16.6%), extraversion (standardized indirect effect = 0.152 [0.005, 0.362], MP = 32.8%), and conscientiousness (standardized indirect effect = 0.150 [0.005, 0.489], MP = 18.5%) (Figure 3A).

Besides, interactions were found between blindness and mobile phone addiction. A significant interaction between blindness and MPAI_WE on extraversion (p_uncorrected_ = 0.015) was observed: only in the blind group, higher MPAI_WE correlated with greater extraversion (t = 2.252, p_uncorrected_ = 0.029) (Figure 3B). Additionally, an interaction was observed between blindness and MPAI_IC on agreeableness (p_uncorrected_ = 0.036), with lower MPAI_IC associated with higher agreeableness in the blind group (t = −2.641, p_uncorrected_ = 0.011) (Figure 3C).

### 3.5 Association between brain imaging phenotypes and personality/blindness

#### 3.5.1 Main effects of personality on brain imaging

**Figure 4:**
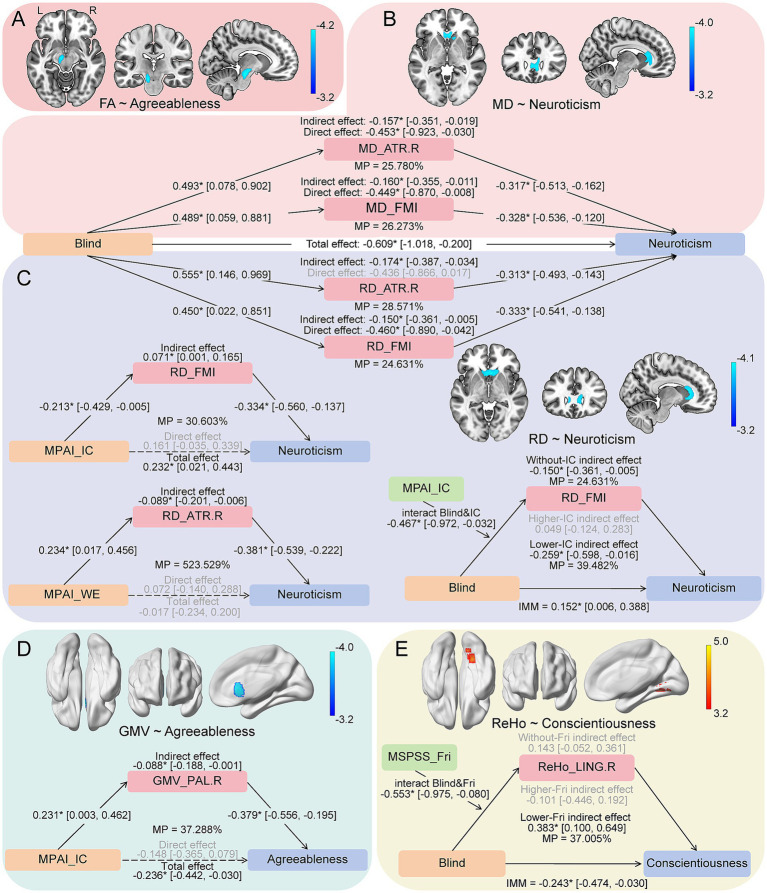
Personality-brain relationships and the potential mediation pathways. Different background colors represent five different brain imaging phenotypes: **(A)** FA, **(B)** MD, **(C)** RD, **(D)** GMV, **(E)** ReHo. For personality-brain correlation analyses, the sample sizes were 84–87 (cases were 43–46). Brain maps represent the whole-brain association between imaging phenotypes and personality traits. Significant brain regions are displayed (voxel-wise *p* < 0.001 uncorrected, cluster-wise *p* < 0.05 FWE corrected), with colors corresponding to the T-values. For mediation analyses, the sample sizes were 77~87 (cases were 42~46). Significance of mediation effects is estimated by 95% percentile confidence intervals using 1,000 bootstraps. Asterisks + solid lines + black font indicate significant pathways, while dashed line with gray font indicate non-significant pathways. All results were adjusted for age and sex, and GMV statistics in personality-brain correlation analyses additionally adjusted for total intracranial volume. MP, mediation proportion; IMM, index of moderated mediation; FA, fractional anisotropy; MD, mean diffusivity; RD, radial diffusivity; GMV, gray matter volume; ReHo, regional homogeneity; ATR. R, right anterior thalamic radiation; FMI, forceps minor; PAL. R, right pallidum; LING. R, right lingual gyrus; MSPSS_Fri, perceived social support from friends; MPAI_WE, withdrawal and escape for mobile phone use; MPAI_IC, inability to control craving for mobile phone use.

In the whole-brain analysis, the main effects of personality on brain imaging phenotypes are shown in Figure 4. Across all participants, agreeableness was negatively correlated with FA in the left corticospinal tract (CST.L) (Figure 4A) and GMV in the right pallidum (PAL.R) (Figure 4D). Neuroticism showed negative associations with MD (Figure 4B) and RD (Figure 4C) in the right anterior thalamic radiation (ATR.R) and forceps minor (FMI). Conscientiousness correlated positively with ReHo in the right lingual gyrus (LING.R) (Figure 4E). No relationships were observed for openness, AD, or ALFF (Supplementary Table 4).

#### 3.5.2 Interactions between blindness and personality on brain imaging

**Figure 5:**
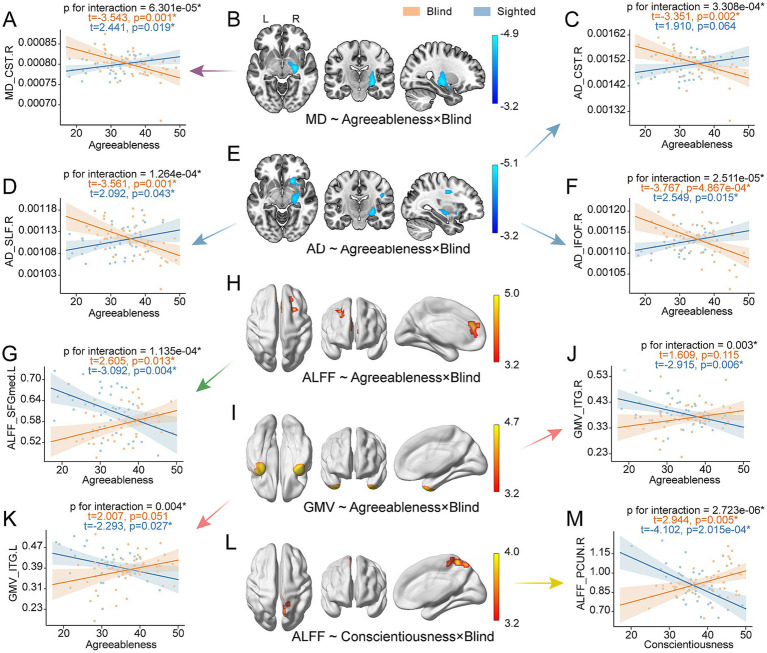
Interaction between personality traits and blindness on brain phenotypes. The sample sizes were 84~87 (cases were 43~46). All results were adjusted for age and sex, and GMV statistics additionally adjusted for total intracranial volume. **(B, E, H, I, L)** Brain maps show the regions where the interaction between blindness and personality traits on brain imaging phenotypes. Significant brain regions are displayed (voxel-wise *p* < 0.001 uncorrected, cluster-wise *p* < 0.05 FWE corrected), with colors corresponding to the T-values of the correlation analyses. **(A, C, D, F, G, J, K, M)** The scatter plots illustrate the *post hoc* relationships between brain imaging phenotypes and personality traits in blind and sighted individuals, respectively. Asterisks represent statistical significance (puncorrected < 0.05). MD, mean diffusivity; AD, axial diffusivity; GMV, gray matter volume; ALFF, amplitude of low frequency fluctuation; CST.R, right corticospinal tract; SLF.R, right superior longitudinal fasciculus; IFOF.R, right inferior fronto-occipital fasciculus; SFGmed.L, left medial superior frontal gyrus; PCUN.R, right precuneus; ITG.L and ITG.R, left and right inferior temporal gyrus.

Significant interactions between agreeableness and blindness were found across multiple brain regions and modalities, including MD in the right corticospinal tract (CST.R) (Figures 5A,B); AD in the right superior longitudinal fasciculus (SLF.R), CST.R, and right inferior fronto-occipital fasciculus (IFOF. R) (Figures 5C–F); ALFF in the left medial superior frontal gyrus (SFGmed.L) (Figures 5G,H); and GMV in the bilateral inferior temporal gyrus (ITG.L and ITG.R) (Figures 5I–K). Furthermore, a significant interaction between conscientiousness and blindness was observed for ALFF in the right precuneus (PCUN.R) (Figures 5L,M). Among the brain regions showing significant main effects of personality or interactions with blindness, we observed significantly lower ALFF in SFGmed.L for blind participants relative to healthy controls (t = 3.471, pFDR < 0.001), and reduced GMV in ITG.R and increased MD and RD in ATR.R and FMI (P_uncorrected_ < 0.05, pFDR > 0.05) (Supplementary Table 5).

### 3.6 Neural pathways underlying the associations of blindness and contextual environments with personality

The mediating results of the brain on the association between blindness, environments, and personality are summarized in Figure 4. The effects of blindness on neuroticism were significantly mediated by MD and RD in ATR.R and FMI. Standardized indirect effects ranged from −0.15 to −0.17, explaining roughly 25–29% of the overall association. For example, MD in ATR.R showed an standardized indirect effect of −0.157 (95% CI [−0.351, −0.019], MP = 25.780%) (Figures 4B,C). Similarly, the associations of MPAI_IC and MPAI_WE with neuroticism were significantly mediated by RD in these fiber tracts (Figure 4C). Additionally, GMV in PAL. R significantly mediated the association between MPAI_IC and agreeableness (standardized indirect effect = −0.088[−0.188, −0.001], MP = 37.288%) (Figure 4D).

Moderated mediation revealed that MPAI_IC significantly moderated the mediation of the effect of blindness on neuroticism, with RD in FMI as the mediator (IMM = 0.152[0.006, 0.388]) (Figure 4C). The indirect effect was significant only at lower MPAI_IC. Additionally, the mediation effect of ReHo in LING. R on the relationship between blindness and conscientiousness was moderated by MSPSS_Fri (IMM = −0.243[−0.474, −0.030]), with the indirect effect being significant only at lower MSPSS_Fri (Figure 4E).

No significant results were found to support a serial mediation pathway (i.e., blindness → contextual environment → brain → personality).

### 3.7 Sensitivity analysis

No significant differences were found between early and late blindness (Supplementary Tables 6, 7). Neither age of onset nor duration showed significant correlations with personality traits or environmental factors (Supplementary Table 8), indicating stable effects across blindness subtypes.

## 4 Discussion

Neuro-ecology posits that the neural mechanisms underlying cognition and behavior are dynamically shaped by environmental contexts. Building on this perspective, the current study collected both environmental and neuroimaging data to investigate personality traits in blind individuals. Compared with sighted controls, blind participants exhibited higher agreeableness, extraversion, and conscientiousness, alongside lower neuroticism. These differences persisted after controlling for depressive symptoms but were attenuated when accounting for trait anxiety, indicating a close link between blindness-related personality patterns and chronic stress. Moreover, higher social support from friends in blind significantly mediated the relationship between blindness and personality traits, while mobile phone use habits exerted effect by interacting with the blindness. Neuroimaging identified both shared and vision-specific neural correlates of personality. These brain phenotypes mediated the associations between blindness and personality, as well as between mobile phone use and personality traits. Finally, social support and mobile phone self-control moderated the mediation pathways linking blindness, brain organization, and personality. Together, these findings support a neuro-ecological account in which personality differences associated with blindness emerge from the interaction between environmental influences and brain reorganization.

We found that blind individuals showed higher agreeableness, extraversion, and conscientiousness, and lower neuroticism—traits generally linked to emotional balance and prosocial orientation. These findings are consistent with previous studies that reported enhanced self-regulation and social awareness among visually impaired individuals (Coren and Harland, 1995; Klinkosz et al., 2006; Garaigordobil and Bernarás, 2009; Cheng and Furnham, 2017). We speculated that when facing various challenges in daily life, blind individuals may develop greater emotional stability and prosocial behavior. These findings challenge the perspective that sensory loss is more likely to lead to negative psychological consequences (Han et al., 2019; Almidani et al., 2024). Additionally, this study did not identify a significant difference in openness between blind and sighted individuals, which aligns with Garaigordobil’s research on severe visual impairment (Garaigordobil and Bernarás, 2009). This may indicate that blindness does not significantly affect an individual’s open-mindedness or interest in exploring new experiences. It should be noted that most of these consistent studies utilized the Big Five personality traits framework, potentially resolving inconsistencies from studies that employed other scales (Gerc, 2018). However, the heterogeneity across studies may also reflect other factors, such as self-selection bias, cultural influences, mental health and other unmeasured factors. Consistent with this, our results further showed that blindness-related personality traits were significantly associated with both anxiety and depression. Notably, while the association between blindness and personality persisted after controlling for depressive symptoms, it was attenuated when accounting for trait anxiety. These findings indicate that the personality profiles associated with blindness are deeply intertwined with chronic anxiety, consistent with prior research that highlights the complex psychological interplay between personality dimensions and mental health (Kotov et al., 2010; Gupta et al., 2024).

One novel contribution of our study is elucidating how blindness-related environmental factors mediate and moderate the effects of blindness on personality. We found that perceived social support from friends mediated the link between blindness and the positive personality traits of agreeableness, extraversion, and conscientiousness. This suggests that blind individuals may become more dependent on others in both practical and emotional aspects (Raina et al., 2004; Schneider et al., 2010). This dependency encourages them to form closer relationships with friends, who in turn may provide more support for them (Brown, 1999). Consistently, among individuals with disabilities, social support is reported to be positively correlated with agreeableness, extraversion, and conscientiousness (Cai et al., 2023). Furthermore, we identified an interactive effect of blindness and mobile phone use on personality profiles. The interaction effects suggest that for blind individuals, mobile phones are not merely a tool but a unique part of their compensatory toolkit. Due to their visual disability, blind individuals may rely more on their mobile phones (Khan and Khusro, 2021) and are less likely distracted by visual stimuli (Giraud et al., 2018). Research has shown that internet addiction is negatively correlated with agreeableness in individuals with disabilities (Pino et al., 2024). We observed that better self-control over phone use (lower phone addiction) in blind individuals was associated with higher agreeableness, possibly because it facilitates more meaningful real-world social interactions. Additionally, we observed that blind individuals may more frequently use mobile phones as a way to escape negative events in real life (Lin et al., 2018). However, this form of escapism may not necessarily be negative. Instead, it may serve as an effective emotion-regulation strategy that allows for greater social engagement in other contexts (Seymour and Lupton, 2004; Shpigelman et al., 2009). These findings suggesting that personality differences in blind individuals may reflect context-dependent shifts rather than intrinsic changes caused by sensory loss.

Neuroimaging analyses revealed both universal and blindness-specific brain-personality relationships, providing insights into neural mechanisms underlying personality development. Universal relationships involved key white matter tracts and subcortical structures previously implicated in personality research. For example, negative associations between neuroticism and diffusivity measures in the anterior thalamic radiation and forceps minor align with prior findings linking these tracts to emotional regulation (Kubota et al., 2012; Xu and Potenza, 2012; Bjørnebekk et al., 2013; Moghaddam et al., 2020). Similarly, the relationship between pallidum volume and agreeableness is consistent with evidence for basal ganglia involvement in social behavior mechanisms (Turner et al., 2016; Aloi et al., 2023; Guo et al., 2024). Blindness-specific interactions prominently involved visual-associated regions including the inferior temporal gyrus, superior longitudinal fasciculus, and inferior fronto-occipital fasciculus. These findings suggest that visual deprivation leads to reorganization of personality-related networks, potentially repurposing visual processing circuits for social-cognitive functions (Leporé et al., 2010; Bagga et al., 2014; Ricciardi et al., 2014; Hirsch et al., 2015; Sripada et al., 2015; Debrabant et al., 2016; Mizuguchi et al., 2017). The involvement of regions such as the precuneus and medial superior frontal gyrus—areas implicated in self-referential processing and social cognition—indicates that blindness may enhance personality traits partly involving strengthened introspective and social-cognitive networks.

The mediation analyses identified multiple neural pathways linking blindness to personality changes, with white matter alterations in vision-related tracts, such as the ATR and FMI (Wang et al., 2013; Frezzotti et al., 2014; Sripada et al., 2015; Tu et al., 2018), acting as key mediators. These tracts are also critically involved in emotional control and regulation. Previous studies have shown that reduced white matter integrity in ATR (Kubota et al., 2012) and agenesis of the corpus callosum (Paul et al., 2021) are associated with alexithymia. Individuals with corpus callosum agenesis demonstrate reduced sensitivity to emotional categories of stimuli, particularly those with negative valence (Paul et al., 2006). This study found that reduced diffusivity in the ATR and FMI mediated the relationship between blindness and lower neuroticism, suggesting that structural modifications in these emotion-regulation circuits may contribute to greater emotional stability. These pathways might represent compensatory reorganization, in which impaired visual processing circuits are repurposed to support improved emotional regulation and social cognition.

Finally, our moderated mediation analyses revealed that environmental factors significantly moderate these brain-mediated personality changes. We found that self-control over mobile phone use and the level of social support act as key regulators in these pathways. For example, self-control over mobile phone use moderates the relationship between blindness, RD of FMI, and neuroticism. The mediating effect of the FMI was most significant in blind individuals characterized by better control over their phone use. This suggests that good self-control allows them to better utilize the brain’s regulatory mechanisms to reduce the impact of negative emotions, reflected in lower neuroticism. Additionally, individuals may also use their phones to escape from real-world pressures by adverse events, which also serves to alleviate emotional distress and lower neuroticism, but through an external coping strategy rather than internal regulation. Similarly, we found that the right lingual gyrus mediated the link between blindness and conscientiousness, but this effect was only significant when individuals reported low social support. Collectively, these findings highlight a dynamic interplay between internal neural changes (e.g., brain reorganization) and external environmental resources (e.g., behavioral and social factors), reflecting a neuro-ecological perspective on personality development following blindness.

Several limitations should be considered, particularly given the inherent complexity of modeling the interplay among blindness, environmental factors, neurobiology, and personality. First, the cross-sectional design means that all variables except blindness status were assessed concurrently, precluding causal inferences regarding these multifaceted neuro-ecological pathways. Second, our relatively small sample size, combined with the heterogeneity of blindness etiology and unmeasured variables (e.g., education and detailed etiological history), may limit statistical power and generalizability while increasing the risk of selection bias and residual confounding. Third, our reliance on subjective self-reports for environmental and personality metrics may introduce bias. Notably, the MPAI is not specifically validated for the non-visual mobile phone usage patterns of blind populations, rendering those specific findings exploratory. Finally, the regions of interest (ROIs) used in our mediation analyses were derived from personality main effects within the same dataset. Because sample size constraints prevented split-half cross-validation, this non-independent selection risks circularity and inflated effect size estimates. To address these limitations, future research should adopt longitudinal designs (e.g., prospective cohorts) and incorporate informant reports alongside objective behavioral measures. Ultimately, validating these neural mechanisms requires larger, preregistered studies utilizing independent samples.

In conclusion, this study indicates that blindness is associated with personality changes through both neural reorganization and environmental modification, consistent with a neuro-ecological account. Higher agreeableness, extraversion, and conscientiousness, together with lower neuroticism, suggest context-dependent personality trait shifts linked to neural reorganization rather than a deficit. Furthermore, these personality profiles are closely intertwined with the management of long-term anxiety. Together, these findings provide new evidence for the joint contribution of environmental experience and brain reorganization to personality, highlighting potential neural markers for understanding resilience in the absence of vision, and advancing our understanding of psychological well-being in visually impaired populations.

## Data Availability

The raw data supporting the conclusions of this article will be made available by the authors, without undue reservation.

## Footnotes

1
https://www.fil.ion.ucl.ac.uk/spm/software/spm12/

2
http://www.neuro.uni-jena.de/cat12-html

3
https://fsl.fmrib.ox.ac.uk/fsldownloads_registration

4
http://rfmri.org/DPARSF

5
https://www.fil.ion.ucl.ac.uk/spm/
