# Supplementary material for: How blindness shapes personality: a neuro-ecological account
Source: Front Neurosci. 2026 Apr 10;20:1759728. doi: 10.3389/fnins.2026.1759728 (PMC13106326; doi:10.3389/fnins.2026.1759728)
Supplement: Supplementary file 1 [file Data_Sheet_1.pdf]

How Blindness Shapes Personality: A Neuro-Ecological Account

Xue Zhang[1†], Qiqi Dong[1†], Yang Liu[1, 2†], Jine Xu[1, 2†], Liyuan Lin[1], Yi Ji[1], Yu Zhang[1,3], Zhen Zhao[1], Zhongyu Chang[1], Luli Wei[1], Xin Li[1], Yun Luo[1], Xinglong Fu[1], Yu Liu[1], Chong Liu[1,2*], Hao Ding[1,4*], Wen Qin[1*]

[1]Department of Radiology, Tianjin Key Lab of Functional Imaging, Tianjin Institute of Radiology and State Key Laboratory of Experimental Hematology, Tianjin Medical University General Hospital, Tianjin 300052, China

[2]Department of Radiology Baoding No.1 Central Hospital, Changchengbei street No. 320, Lianchi District, Baoding 071032, China

[3]Department of Radiology, Beijing Friendship Hospital, Capital Medical University, Beijing 100050, China

[4]School of Medical Imaging, Division of Medical Technology, Tianjin Medical University, Tianjin, China

Supplementary Material

Supplementary Figures

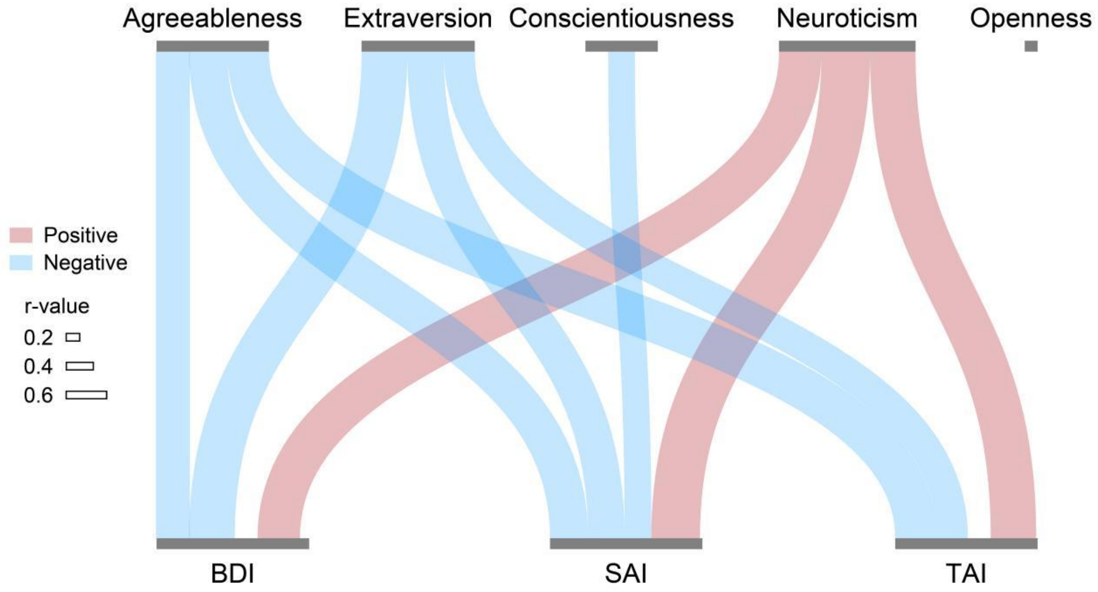

Supplementary Figure 1. Partial correlations between personality traits and measures of depression and anxiety in the blind group. Sample size = 46. The results were adjusted for age and sex. Edge

widths reflect the strength of the correlation coefficients, with red edges indicating positive correlations and blue edges indicating negative correlations. Only significant edges (pFDR < 0.05) are shown. Abbreviations: BDI, beck depression inventory; SAI, state anxiety index; TAI, trait anxiety index.

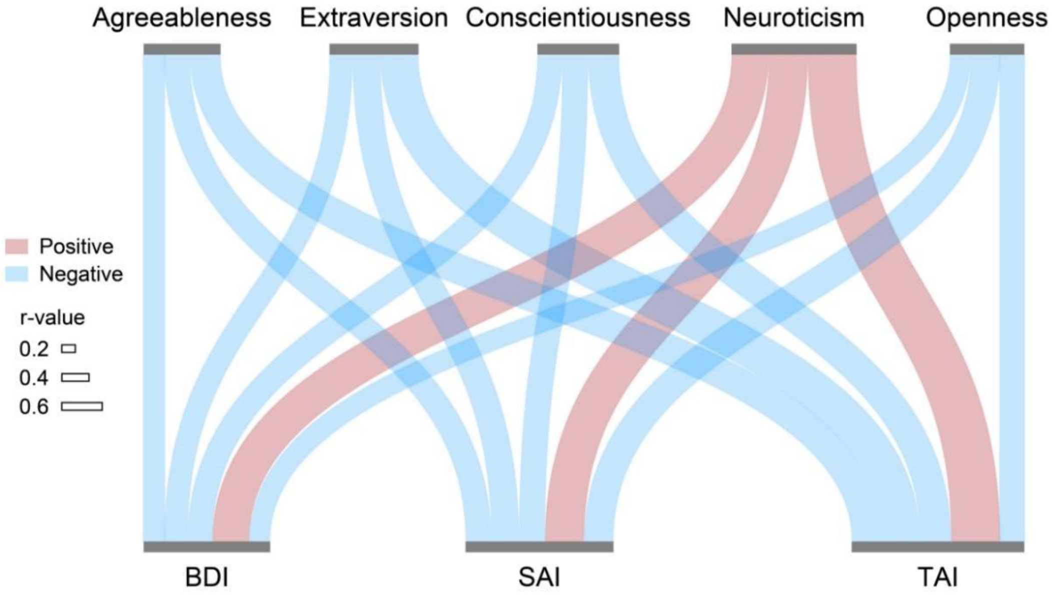

Supplementary Figure 2. Partial correlations between personality traits and measures of depression and anxiety in the sighted group. Sample size = 41. The results were adjusted for age and sex. Edge widths reflect the strength of the correlation coefficients, with red edges indicating positive correlations and blue edges indicating negative correlations. Only significant edges (pFDR < 0.05) are shown. Abbreviations: BDI, beck depression inventory; SAI, state anxiety index; TAI, trait anxiety index.

Supplementary Tables

Table S1. Demographic information of blind subjects

PID	Sex	Age (years)	Age of onset (years)	Duration (years)	Etiology
1	male	45	0	45	-
2	female	36	0	36	eyes stunting
3	female	38	17	21	-
4	male	29	0	29	-
5	female	23	0	23	-
6	female	34	21	13	-
7	female	36	8	28	malignant tumors of the eye
8	female	35	7	28	-
9	female	41	0	41	-
10	female	41	5	36	-
11	female	41	16	25	fundus oculi disease
12	male	43	34	9	fundus oculi disease
13	male	59	25	34	congenital cataract
14	male	51	14	37	eye trauma
15	female	48	25	13	amotio retinae
16	male	54	13	41	amotio retinae
17	male	35	0	35	-
18	male	49	0	49	-
19	male	45	0	45	
20	male	42	12	30	-
21	male	46	0	46	-
22	female	39	0	39	-
23	female	58	2	56	-
24	male	53	13	40	eye trauma
25	male	54	30	24	-
26	female	26	2	24	glaucoma
27	male	39	10	29	hypoplasia of the eye
28	male	51	34	17	eye blast
29	female	55	34	21	retinal detachment, congenital
30	female	64	1	63	keratohelcosis
31	female	28	0	28	-
32	male	32	0	32	-
33	female	49	30	19	-
34	male	44	33	11	-
35	female	40	3	37	adverse drug reactions in eyes
36	male	56	12	44	amotio retinae

37	male	54	25	19	-
38	male	53	3	50	eye trauma
39	female	46	0	46	-
40	female	41	39	2	-
41	male	35	14	21	-
42	male	46	30	16	-
43	female	55	13	42	-
44	female	46	0	46	-
45	male	58	21	37	-
46	female	62	40	22	-

Table S2. Differences in environmental factors between blind and sighted individuals

Exposure	Blind(M±SD)	Sighted(M±SD)	Effect Size (95% CI)	Statistic	pFDR
MPAI_IC	11.043±3.712	14.127±5.483	0.28 (0.05, 0.47)	Z=-2.539	0.037*
MPAI_WE	11.261±3.708	7.553±2.992	0.47 (0.27, 0.63)	Z=4.300	0.010*
MPAI_AL	9.022±4.019	9.607±3.929	-0.14 (-0.58, 0.29)	t=-0.657	0.513
MPAI_PL	7.000±3.011	7.420±2.974	0.09 (0.00, 0.30)	Z=-0.867	0.429
MSPSS_Fam	21.742±6.755	21.906±4.412	0.11 (0.00, 0.32)	Z=0.950	0.428
MSPSS_Fri	23.778±4.597	21.699±4.672	0.29 (0.08, 0.48)	Z=2.604	0.037*
MSPSS_SO	22.883±5.460	21.998±3.976	0.22 (0.02, 0.42)	Z=1.949	0.100
PSQI	6.087±3.788	4.892±3.007	0.14 (0.01, 0.34)	Z=1.241	0.306
TFEQ	39.273±8.258	41.639±6.489	0.21 (0.03, 0.41)	Z=-1.883	0.100
CSSS	56.851±11.226	61.994±10.779	-0.46 (-0.92, 0.00)	t=-2.006	0.100

Notes: * pFDR < 0.05. Sample size = 84 (cases = 46; MPAI), sample size = 80 (cases = 45; MSPSS), sample size = 83 (cases = 46; PSQI and TFEQ), and sample size = 79 (cases = 46; CSSS). The results were adjusted for age and sex. Abbreviations: M±SD, mean±standard deviation; MPAI_IC, inability to control craving for mobile phone use; MPAI_WE, withdrawal and escape for mobile phone use; MPAI_AL, anxiety and loss for mobile phone use; MPAI_PL, productivity loss for mobile phone use; MSPSS_Fam, perceived social support from family; MSPSS_Fri, perceived social support from friends; MSPSS_SO, perceived social support from significant others; PSQI, sleep quality; TFEQ, three-factor eating; CSSS, subjective social status.

Table S3. Differences in personality traits between blind and sighted individuals after controlling for depression and anxiety

Covariates	Personality	Statistic	pFDR
BDI	Neuroticism	Z=-4.464	< 0.001*
	Agreeableness	t=4.121	< 0.001*
	Conscientiousness	Z=3.920	< 0.001*
	Extraversion	Z=3.197	0.006*
SAI	Neuroticism	Z=-2.304	0.057
	Agreeableness	Z=2.168	0.064
	Conscientiousness	t=3.161	0.009*
	Extraversion	t=0.950	0.394
TAI	Neuroticism	Z=-0.795	0.456
	Agreeableness	Z=1.122	0.349
	Conscientiousness	t=2.157	0.064
	Extraversion	Z=0.272	0.786
BDI + SAI + TAI	Neuroticism	Z=-1.718	0.137
	Agreeableness	Z=0.400	0.736
	Conscientiousness	t=1.665	0.142
	Extraversion	Z=1.148	0.349

Notes: * pFDR < 0.05. The results were adjusted for age and sex. Sample size = 87 (cases = 46).
Abbreviations: BDI, beck depression inventory; SAI, state anxiety index; TAI, trait anxiety index.

Table S4. Statistically significant brain regions associated with personality

Cluster	Cluster size	Anatomic	x	y	z	Statistics (t)
**Main effect**						
FA - Agreeableness						
Cluster 1	203	CST.L	-10	-22	-18	-4.217
RD - Neuroticism						
Cluster 1	740	ATR.R	16	16	8	-3.869
		FMI	6	22	6	-3.986
MD - Neuroticism						
Cluster 1	385	ATR.R	14	14	6	-3.828
		FMI	4	26	0	-3.996
GMV - Agreeableness						
Cluster 1	1518	PAL.R	15	9	0	-4.004
ReHo - Conscientiousness						
Cluster 1	62	LING.R	24	-57	-3	5.368
**Interactive effect**						
AD - Agreeableness						
Cluster 1	481	CST.R	22	-18	-6	-4.008
		IFOF.R	32	-8	-8	-3.826
Cluster 2	354	SLF.R	34	-8	34	-4.090
Cluster 3	223	IFOF.R	22	20	-4	-4.972
MD - Agreeableness						
Cluster 1	538	CST.R	22	-16	-6	-4.309
GMV - Agreeableness						
Cluster 1	2064	ITG.R	41	5	-44	5.053
Cluster 2	2061	ITG.L	-32	2	-41	4.824
ALFF - Agreeableness						
Cluster 1	266	SFGmed.L	-15	42	24	4.574
ALFF - Conscientiousness						
Cluster 1	115	PCUN.R	6	-48	60	4.968

Notes: The sample sizes were 84 ~ 87 (cases were 43 ~ 46). All results were adjusted for age and sex, and GMV statistics additionally adjusted for total intracranial volume. Abbreviations: FA, fractional anisotropy; RD, radial diffusivity; MD, mean diffusivity; AD, axial diffusivity; GMV, gray matter volume; ReHo, regional homogeneity; ALFF, amplitude of low frequency fluctuation; CST.L and CST.R, left and right corticospinal tract; ATR.R, right anterior thalamic radiation; FMI, forceps minor; PAL.R, right pallidum; LING.R, right lingual gyrus; IFOF.R, right inferior fronto-occipital fasciculus; SLF.R, right superior longitudinal fasciculus; ITG.L and ITG.R, left and right inferior temporal gyrus; SFGmed.L, left medial superior frontal gyrus; PCUN.R, right precuneus.

Table S5. Differences in personality-related brain regions between blind and sighted group

Anatomic region	Blind(M±SD)	Sighted(M±SD)	Statistic	$p_{uncorrected}$
**Main effect**				
FA_CST.L	0.509±0.021	0.515±0.025	t=-1.339	0.184
RD_ATR.R	8.311e-04±1.048e-04	7.748e-04±8.413e-05	t=2.673	0.009*
RD_FMI	9.430e-04±1.772e-04	8.678e-04±1.425e-04	t=2.137	0.035*
MD_ATR.R	9.132e-04±7.817e-05	8.763e-04±5.968e-05	t=2.356	0.021*
MD_FMI	1.102e-03±1.037e-04	1.054e-03±7.768e-05	t=2.332	0.022*
GMV_PAL.R	0.463±0.048	0.471±0.057	t=-0.920	0.360
ReHo_LING.R	0.960±0.107	0.932±0.107	t=1.398	0.166
**Interactive effect**				
AD_CST.R	1.503e-03±5.693e-05	1.499e-03±4.995e-05	Z=-0.451	0.652
AD_IFOF.R	1.130e-03±4.358e-05	1.125e-03±3.321e-05	t=0.234	0.815
AD_SLF.R	1.114e-03±4.449e-05	1.106e-03±4.112e-05	t=0.577	0.566
MD_CST.R	7.986e-04±3.541e-05	7.975e-04±2.461e-05	Z=0.349	0.727
GMV_ITG.R	0.370±0.055	0.394±0.054	Z=2.052	0.040*
GMV_ITG.L	0.381±0.069	0.403±0.059	t=-1.473	0.144
ALFF_SFGmed.L	0.574±0.049	0.617±0.066	t=-3.471	< 0.001**
ALFF_PCUN.R	0.938±0.128	0.911±0.142	t=1.126	0.263

Notes: * $p_{uncorrected} < 0.05$, ** $p_{FDR} < 0.05$~~, corrected by false discovery rate (FDR)~~. The sample sizes were 84 ~ 87 (cases were 43 ~ 46). All results were adjusted for age and sex, and GMV statistics additionally adjusted for total intracranial volume. Abbreviations: M±SD, mean±standard deviation; FA, fractional anisotropy; RD, radial diffusivity; MD, mean diffusivity; AD, axial diffusivity; GMV, gray matter volume; ReHo, regional homogeneity; ALFF, amplitude of low frequency fluctuation; CST.L and CST.R, left and right corticospinal tract; ATR.R, right anterior thalamic radiation; FMI, forceps minor; PAL.R, right pallidum; LING.R, right lingual gyrus; IFOF.R, right inferior fronto-occipital fasciculus; SLF.R, right superior longitudinal fasciculus; ITG.L and ITG.R, left and right inferior temporal gyrus; SFGmed.L, left medial superior frontal gyrus; PCUN.R, right precuneus.

Table S6. Differences in personality and environments among the early blind (onset ≤ 6 years), late blind (onset > 6 years) and sighted group

	Statistics	$p_{uncorrected}$	(I)group	(J)group	Mean difference (I-J)	pFDR
Neuroticism	H = 8.331	0.016*	early	late	1.308	0.607
			early	sighted	-5.299	0.082
			late	sighted	-6.606	0.021*
Agreeableness	F = 7.135	0.001*	early	late	-2.606	0.194
			early	sighted	3.624	0.076
			late	sighted	6.230	0.001*
Conscientiousness	H = 13.528	0.001*	early	late	0.126	0.958
			early	sighted	4.808	0.005*
			late	sighted	4.682	0.005*
Extraversion	H = 4.407	0.110	-	-	-	-
MPAI_IC	H = 6.549	0.038*	early	late	0.341	0.748
			early	sighted	-2.851	0.103
			late	sighted	-3.192	0.056
MPAI_WE	H = 18.660	< 0.001*	early	late	0.564	0.683
			early	sighted	4.044	0.001*
			late	sighted	3.480	0.001*
MSPSS_Fri	H = 9.027	0.011*	early	late	-2.194	0.201
			early	sighted	0.914	0.253
			late	sighted	3.108	0.008*

Notes: * $p < 0.05$. In personality comparison, the sample sizes were 20 (early blind), 26 (late blind) and 41 (sighted). In environments comparison, the sample sizes for MPAI were 20 (early blind), 26 (late blind) and 38 (sighted); for MSPSS were 19 (early blind), 26 (late blind) and 35 (sighted). The results were adjusted for age and sex. P values from the overall three-group comparisons (ANCOVA or Kruskal–Wallis test) were uncorrected. P values from the three post hoc pairwise comparisons were adjusted using the FDR correction. Abbreviations: MPAI_IC, inability to control craving for mobile phone use; MPAI_WE, withdrawal and escape for mobile phone use; MSPSS_Fri, perceived social support from friends.

Table S7. Differences in personality and environments among the early blind (onset < 10 years), late blind (onset ≥ 10 years) and sighted group

	Statistics	$p_{uncorrected}$	(I)group	(J)group	Mean difference (I-J)	pFDR
Neuroticism	H = 8.498	0.014*	early	late	1.486	0.511
			early	sighted	-5.263	0.081
			late	sighted	-6.748	0.019*
Agreeableness	F = 7.685	0.001*	early	late	-3.212	0.106
			early	sighted	3.421	0.083
			late	sighted	6.633	0.001*
Conscientiousness	H = 13.541	0.001*	early	late	0.271	0.901
			early	sighted	4.878	0.004*
			late	sighted	4.607	0.004*
Extraversion	H = 4.446	0.108	-	-	-	-
MPAI_IC	H = 6.792	0.034*	early	late	0.622	0.556
			early	sighted	-2.719	0.123
			late	sighted	-3.341	0.043*
MPAI_WE	H = 18.493	< 0.001*	early	late	0.141	0.997
			early	sighted	3.798	0.001*
			late	sighted	3.657	0.001*
MSPSS_Fri	H = 9.899	0.007*	early	late	-2.203	0.116
			early	sighted	1.007	0.268
			late	sighted	3.210	0.005*

Notes: * $p < 0.05$. In personality comparison, the sample sizes were 22 (early blind), 24 (late blind) and 41 (sighted). In environments comparison, the sample sizes for MPAI were 22 (early blind), 24 (late blind) and 38 (sighted); for MSPSS were 21 (early blind), 24 (late blind) and 35 (sighted). The results were adjusted for age and sex. P values from the overall three-group comparisons (ANCOVA or Kruskal–Wallis test) were uncorrected. P values from the three post hoc pairwise comparisons were adjusted using the FDR correction. Abbreviations: MPAI_IC, inability to control craving for mobile phone use; MPAI_WE, withdrawal and escape for mobile phone use; MSPSS_Fri, perceived social support from friends.

Table S8. The correlation between the age of onset of blindness, the duration of blindness, and personality and environments

	Age of onset of blindness		Duration of blindness	
	r	$p_{uncorrected}$	r	$p_{uncorrected}$
Neuroticism	-0.259	0.082	0.118	0.434
Agreeableness	0.278	0.061	-0.108	0.474
Conscientiousness	0.151	0.315	-0.069	0.647
Extraversion	0.131	0.386	-0.055	0.719
MPAI_IC	-0.092	0.542	-0.092	0.542
MPAI_WE	-0.082	0.587	0.052	0.733
MSPSS_Fri	0.193	0.204	-0.197	0.194

Notes: r = Correlation Coefficient. In personality correlation, the cases were 46. In environments correlation, the cases for MPAI were 46; for MSPSS were 45. Abbreviations: MPAI_IC, inability to control craving for mobile phone use; MPAI_WE, withdrawal and escape for mobile phone use; MSPSS_Fri, perceived social support from friends.
